# Time to Move On: Redefining Chest Pain Outcomes

**DOI:** 10.1161/JAHA.119.012542

**Published:** 2019-06-11

**Authors:** Michael B. Weinstock, Nathan M. Finnerty, Michael Pallaci

**Affiliations:** ^1^ Department of Emergency Medicine Adena Emergency Medicine Residency Chillicothe OH; ^2^ Department of Emergency Medicine Wexner Medical Center at The Ohio State University Columbus OH; ^3^ Department of Emergency Medicine Intermountain Medical Center Salt Lake City UT; ^4^ Ohio University Heritage College of Osteopathic Medicine Chillicothe OH

**Keywords:** acute cardiac care, acute coronary syndrome, adverse events complication, risk score, risk stratification, Quality and Outcomes, Risk Factors, Cost-Effectiveness, Acute Coronary Syndromes

Chest pain is the second most common presenting symptom to emergency departments (EDs), accounting for >7 million visits in the United States each year.^1^ However, as few as 10% of patients presenting to the ED with chest pain will ultimately be diagnosed with acute coronary syndrome (ACS).^2^ ACS can be a challenging diagnosis in the ED as the initial history, physical examination, and ECG alone can neither confirm nor exclude the diagnosis.^2^, ^3^

Although there have been many clinical decision rules for chest pain evaluation, the HEART score (history, EGG, age, risk factors, troponin) has come to the forefront of emergency medicine practice, as it is the only model to be evaluated by multiple independent research groups in both validation and clinical impact studies and has outperformed alternate prediction models in comparison studies.^2^, ^3^

It was designed to assess patients presenting to the ED with possible ACS and identify those at low risk for adverse events who may be suitable for discharge.^4^ However, the HEART score can also be used to justify hospital admission for those deemed to be moderate to high risk for a major adverse cardiac event (MACE), which may lead to increased testing and/or intervention.

But is MACE an appropriate outcome? And is the timeline relevant to a decision at the beside in the ED? A MACE outcome is a composite of acute myocardial infarction (MI), percutaneous coronary intervention, coronary artery bypass graft surgery, and all‐cause death. Composite outcome measures, such as MACE, result in higher event rates and have been used when other serious events (missed MI or death) are relatively rare. Clinical trials designed with composite end points subsequently require fewer patients, are less costly, and can be completed more quickly. Death and acute MI are important measures to assess in composite, but including revascularization (percutaneous coronary intervention and coronary artery bypass graft surgery) as outcome measures may imply that an adverse event has occurred, although appropriate emergent care has been provided. In addition, the MACE outcome is measured at 4–6 weeks, an unusual timeline in the ED disposition decision. This begs the question: does an increased risk of MACE at 4–6 weeks justify immediate hospitalization or emergent intervention?

The recommendation to hospitalize after ED evaluation has typically been focused on immediate risk (ie, will the patient experience an adverse event over the next few days, or will the patient be able to safely complete his/her evaluation as an outpatient?). Should a clinical decision rule based on outcomes at 4–6 weeks be the basis of our disposition decision? The short‐term risk for an adverse event after a negative ED chest pain evaluation is extremely low.^5^ Even after an ischemic event, such as a non–ST‐segment–elevation MI, the 6‐month risk of sudden cardiac death is <1%; extrapolating to a 5‐day period of time, the risk is 0.02%.^6^ Furthermore, hospitalization is not without risk and should be avoided when possible.^7^

The American College of Emergency Physicians (ACEP) just published guidelines confirming an acceptable miss rate of MACE of 1–2%, but data have shown that the acceptable miss rate for most emergency physicians is <1%.^3^, ^8^, ^9^ In the absence of consensus, variations in provider practice, risk aversion, malpractice concerns, the fear of poor patient outcome or missed diagnosis, and/or the fear of loss of respect from colleagues may drive emergency physicians to increase resource use and hospitalization.^10^

In a basic model of diagnostic uncertainty, testing may continue until the risks or harms of the test are higher than those of the disease in question. Given the inherent risks and potential harms of hospitalization, outpatient management after a negative ED evaluation should be the mainstay of care, and hospitalization should be the exception. As such, the role of the ED practitioner should include ruling out an acute cardiac event and referring the patient for appropriate follow‐up. This does not exclude the need for thorough assessment of alternative emergent diagnoses that may present with chest pain, such as aortic dissection, pulmonary embolism, pericardial tamponade, cholangitis, pneumonia, or malignancy. However, when such diagnoses have been objectively determined to be unlikely, the focus returns to the disposition of the patient presenting to the ED with chest pain and without an acute cardiac event.

Consider the following case scenarios:

A 65‐year‐old man with a history of hypertension and coronary artery disease presents to his cardiologist with 2 weeks of intermittent, exertional chest pressure and dyspnea. An ECG shows nonspecific findings. His HEART score is 6. The patient is scheduled for a cardiac catheterization the following week. Significant atherosclerotic disease is identified, and revascularization is performed. Most would agree that his evaluation and management were patient centered, expedient, and successful.

Now consider the same patient presentation, only this time in the ED. He has the same history, the same ECG, normal vital signs, and negative serial troponin assays. His HEART score is still 6, placing him at moderate‐risk for a MACE in the next 4–6 weeks. In our current healthcare environment, the patient would likely be admitted to the hospital or placed in an observation unit. But does this make him safer? In both scenarios, he undergoes revascularization, but in the latter, had he been discharged from the ED, his revascularization would have been deemed a missed MACE. This is a contributing factor to patients being exposed to the risks and costs inherent to hospitalization. Does the setting in which care is provided justify these additional risks and healthcare costs?

In ACS, early invasive treatment (within 24–48 hours), with cardiac catheterization and appropriate revascularization, remains the preferred treatment for patients with unstable angina or non–ST‐segment–elevation MI when prohibitive comorbidities are absent.^11^ Percutaneous coronary intervention for stable angina, even with modest amounts of ischemia, has not been shown to reduce death, nonfatal MI, unplanned revascularization, or angina.^11^ Without evidence‐based support for urgent revascularization in the absence of objective findings of myocardial ischemia, infarction, or unstable angina, the outcome of percutaneous coronary intervention or coronary artery bypass graft surgery within the following 4–6 weeks should not be included in the ED disposition decision. However, non–ST‐segment–elevation MI identified in follow‐up and leading to revascularization could be included in the outcome measure.

We propose that when studying ED patients with chest pain, investigators consider using an end point that is more relevant to the disposition decision faced by the ED practitioner and his/her patient. A clinically relevant adverse cardiac event (CRACE) is the composite of life‐threatening arrhythmia, ST‐segment–elevation MI, cardiac or respiratory arrest, or death, which occurs during hospitalization.^5^ Although narrowing the composite outcome measure may challenge future clinical trials in comparison to MACE, redefining what is considered an adverse event would enable us to improve our ability to educate, engage, and safely discharge more patients after the ED evaluation of chest pain. Without appropriately defined outcomes, translation to the bedside is interrupted, variation in practice prevails, and substandard care is rendered. Adopting a clinically relevant adverse cardiac event, as opposed to a MACE, would improve care and reduce healthcare use.

## Disclosures

None.
